# Successful Urgent TAVI for Critical Aortic Valve Stenosis after ECMO Implantation

**DOI:** 10.3390/medicina58030356

**Published:** 2022-02-28

**Authors:** Ya-Lei Niu, Nicola Patrick Mayr, Yin-Hwa Chen, Hsiao-Hwang Chang, Shi-Pu Wang, Hung-Yu Lin, Ching-Chou Pai

**Affiliations:** 1 Department of Cardiology, Chang Bing Show Chwan Memorial Hospital, Changhua 505, Taiwan; niuyalei@gmail.com (Y.-L.N.); wangshihpu@gmail.com (S.-P.W.); 2 Institut für Anästhesiologie, Deutsches Herzzentrum München, Technical University Munich, 80333 Munich, Germany; patrick.mayr@tum.de; 3 Department of Cardiology, Taipei Veterans General Hospital, Taipei 112, Taiwan; yhchen@vghtpe.gov.tw; 4 Department of Cardiac Surgery, Taipei Veterans General Hospital, Taipei 112, Taiwan; do154@yahoo.com.tw; 5 Research Assistant Center, Show Chwan Memorial Hospital, Changhua 500, Taiwan; linhungyu700218@gmail.com; 6 Department of Cardiac Surgery, Show Chwan Memorial Hospital, Changhua 500, Taiwan

**Keywords:** transcatheter aortic valve implantation, extracorporeal membranous oxygenation, aortic valve stenosis

## Abstract

Transcatheter aortic valve implantation (TAVI) has evolved to be the treatment of choice for patients with severe aortic stenosis and high perioperative risk. Cardiogenic shock is one of the most severe complications during the TAVI procedure, especially as the prognosis of cardiogenic shock secondary to aortic stenosis is very poor. This situation can be challenging, while extracorporeal membranous oxygenation (ECMO) can be a treatment option. Here, we reported on an 88-year-old female patient who had been diagnosed as non-ST-elevation myocardial infarction (NSTEMI) and critical aortic valve stenosis (AS) with a logistic Euroscore of 25%. Percutaneous coronary angioplasty (PCI) was performed smoothly and developed tachy-brady arrhythmia of atrial fibrillation then cardiac arrest at the beginning of the TAVI procedure. A v-a ECMO was installed at her left femoral side. Afterward, the TAVI procedure was completed accordingly; her consciousness recovered and Levosimendan therapy enhanced her left-ventricular ejection fraction (LVEF) from 22% to 40%. Five days after TAVI, ECMO was replaced by intra-aortic balloon pumping (IABP) and it was removed 3 days later. A minor complication of this therapy, e.g., muscular weakness in her left leg, was noted. The patient underwent rehabilitation for about 2 months, and was discharged from hospital with a wheel chair and clear consciousness. At the 24 month follow-up she was in good recovery and was able to walk upstairs to the second floor again. Our experience suggests that one indication of prophylactic use of ECMO is for patients with an unstable hemodynamic condition.

## 1. Introduction

Transcatheter aortic valve implantation (TAVI) has changed the treatment for high risk patients with severe aortic stenosis. The target population is elderly, frail patients, particularly in intensive care units, to improve their life expectancy and quality. Coronary artery disease is prevalent in about a half of these patients [1]. Cardiogenic shock is one of the most severe complications during the TAVI procedure [2].

Available extracorporeal membraneous oxygenation (ECMO) on-site can be life-saving, but there are still many complications or risks from ECMO. The most optimal timing of ECMO application is still in discussion. More case reports are needed as large scale of trials are not easily designed. Here, we show the treatment of such a patient.

## 2. Case Report

**Figure 1:**
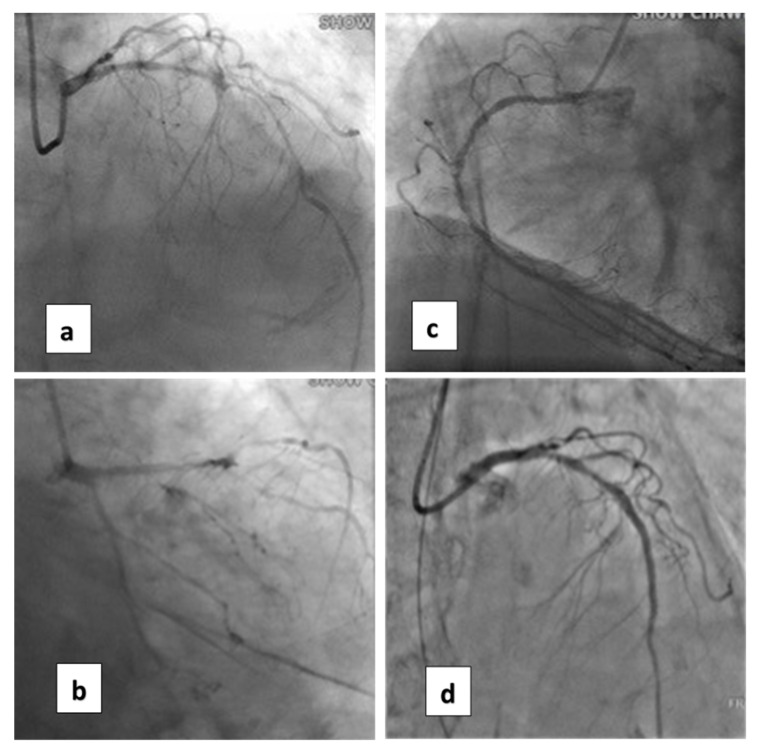
Coronary artery lesions of the patient. (**a**) Cranial view of coronary angiogram (CAG) revealed diffuse severe stenosis of the left anterior descending artery (LAD). (**b**) Caudal view of CAG shows total occlusion of the left circumflex artery (LCX). (**c**) CAG of the right coronary artery (RCA). (**d**) After percutaneous angioplasty (PCI) with two stents for LAD.

An 88-year-old female patient (height = 160 cm, body weight = 59 kg, BMI = 23) had increasing shortness of breath and visited our emergency department. Her past history included bronchial asthma and hypertension with regular treatment at our hospital. Initially, a mild elevated Troponin-I = 1.58 ng/mL (normal value, <0.16) was measured. Coronary angiography revealed three-vessel-disease: left anterior descending artery (LAD), middle-segment with long lesion up to 90% stenosis; left circumflex artery (LCX), distal-segment with chronic total occlusion; and right coronary artery (RCA), middle-segment with 40% tandem lesions (Figure 1a–c). Critical aortic valve stenosis (AS) with a mean pressure gradient (PG) of 43 mmHg was additionally diagnosed. The estimated aortic valve area (Gorlin equation) was 0.6 cm^2^. Her left-ventricular ejection fraction (LVEF) was about 58%.

**Figure 2:**
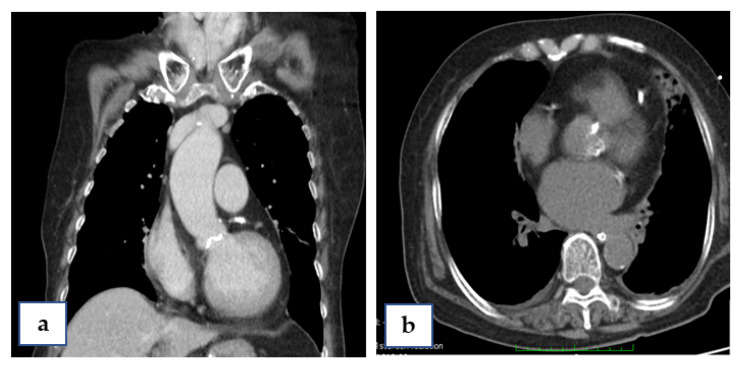
Computed tomography (CT) of the patient, sagittal view (**a**) and coronal view (**b**), showing calcification of the aortic annulus.

**Figure 3:**
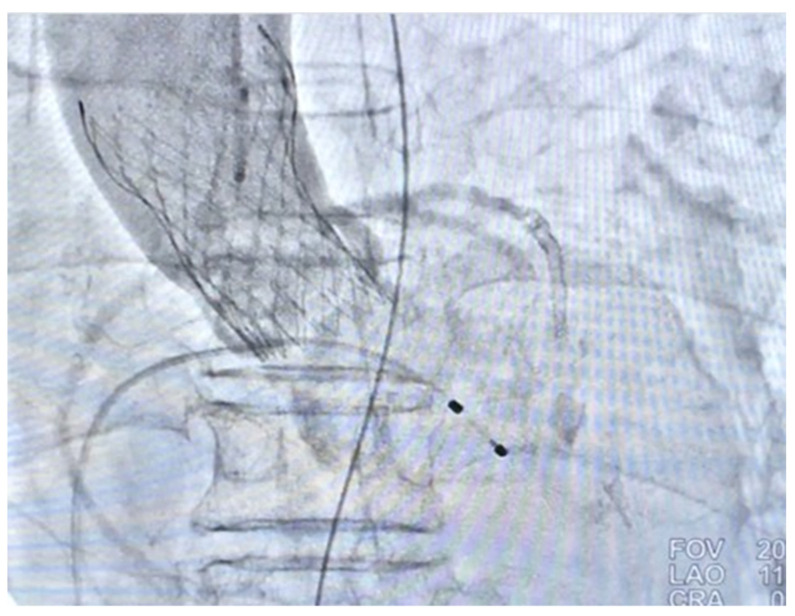
Post-TAVI aorta-gram showing mild aortic regurgitation (AR).

Coronary artery bypass graft (CABG) and surgical aortic valve replacement (SAVR) or percutaneous coronary angioplasty (PCI) and TAVI were suggested to her family. They said they needed a family meeting to discuss it. However, the next day after the coronary angiogram, her dyspnea was more serious and her blood saturation of oxygen was down to 60%. So, she was intubated with mechanical ventilation. Based on her co-morbidities and old age, her logistic Euroscore was 25%, which is high risk (>13%). PCI and TAVI were preferred. PCI was performed next day of intubation, smoothly with two drug-eluting-stents (2.75 mm × 28 mm and 2.5 mm × 28 mm Xience, Abbott, Chicago, IL, USA) in the LAD (Figure 1d). The computed tomography (CT) showed moderate calcification of the aortic valve (Figure 2). Her family requested to wait for an important son coming back from abroad and for her to then receive TAVI. So, she was kept intubated with mild sedation. The patient’s renal function was within the normal limit: blood creatinine was 0.59 mg/dL (normal range 0.50–0.90 mg/dL) before PCI and 0.67 mg/dL after PCI; her hemodynamic condition was stable before and after stent implantation until the night prior to TAVI; four days after PCI, the patient’s vital signs became unstable. Atrial fibrillation (AF) with fluctuated ventricular response and arterial hypotension were noted. Inotropic agent (dopamine 8 μg/kg/min) was given. Upon arrival in the hybrid operation-room, tachycardia atrial fibrillation with about 110 beats per minute bpm was noted. After the induction of general anesthesia, her heart rate dropped to about 60 bpm and she subsequently went into cardiac arrest. Cardiopulmonary resuscitation (CPR) was therefore initiated for about 15 min. A veno-arterial (VA)-ECMO (Capiox, Terumo (Tokyo, Japan; with venous sheath 21 French and arterial sheath 16.5 French) was installed in her left femoral side. Ventricular tachycardia (VT) and ventricular fibrillation (VF) occurred, and 200 joule cardioversion was done twice to restore sinus rhythm. Urgent-TAVI was performed with a 25 mm × 4 cm ZMED2 balloon for pre-dilatation and a 31 mm CoreValve Evolut (Medtronic, Dublin, Ireland) implanted (Figure 3). After the TAVI procedure, she was transferred back to ICU. Despite the sinus rhythm being restored, her LVEF was 22%, which was due to cardiac arrest. We used Levosimendan-therapy and then her LVEF rose to 40%. Pulmonary hemorrhage occurred two days later on ECMO. Her activated partial thromboplastin time (APTT) reached 112.2 s (normal range 24.3–32.7 s). Heparin was held for hours and 10 units of packed red blood cells (RBC) and 4 units and fresh frozen plasma (FFP) were transfused. Then, her APTT was maintained between 50 and 60 s.

Five days after TAVI, ejection fraction of the left ventricle was maintained at 30–40% and her left leg was noted to be motionless. So, ECMO was replaced by intra-aortic balloon pumping (IABP) because the latter can help with heart failure because of its smaller cannula, and the IABP was removed 3 days later. Due to her bronchial asthma, weaning from the ventilator was more difficult. A high dose of steroid (Prednisolone 40 mg QD) was given and the patient was successfully weaned from the ventilator 21 days after the procedure.

As a minor complication of this therapy, we noticed muscular weakness in her left leg, presumably due to restriction of the left leg. The patient underwent rehabilitation for about 2 months, and was discharged from our hospital with a wheel chair and clear conscious. At the 36 month follow-up she was in good recovery and was able to walk upstairs to the second floor again.

## 3. Discussion

Basically, SAVR and TAVI are both suitable options for patients with severe aortic stenosis. The same holds true for coronary artery disease with PCI and CABG as possible options. However, the decision has to be made on the current clinical status and comorbidities. In the presented case, the patient was in cardiogenic shock and cardiac arrest. In such a situation, SAVR and CABG were not an option. Therefore, the decision to implant a v-a ECMO and perform a Rescue-TAVI was made.

When AS patients suffer from hypotension, dopamine may increase the cardiac output and the aortic valve gradient. Instead of dopamine, IABP can be considered.

Sudden death is one of the signs of severe AS patients. Independent predictors associated with a higher risk for sudden death in AS patients includes prior myocardial infarction, body mass index <22, peak aortic jet velocity ≥5 m/s, and LVEF <60% [3]. Our patient manifested non-ST-elevation myocardial infarction about 1 week prior to the TAVI procedure with a LVEF of 53%; these two factors are compatible with the finding of the predictors. As for her cardiac arrest, it seemed to happen after general anesthesia. The muscle relaxant we used was Rocuronium, which is not related to arrhythmia. Drug related anaphylactic shock may have been the cause, or simply AS related cardiac arrest. However, nowadays, nearly all cases are done under conscious sedation.

Concomitant PCI and TAVI have been reviewed by Davide Cao el at al [4]. It is a feasible procedure and may lower the rate of adverse events. However, TAVI may not be reimbursed and needs to be paid by the patient’s family. This was the reason they needed to wait an important son coming back from abroad for the decision. In every TAVI procedure an ECMO should be on-site in case of rescue therapy for severe procedural complications or hemodynamic instability. Trenkwalder et al. reported that the indications of emergent ECMO include ventricular rupture, low cardiac output, bleeding, coronary artery impairment, ventricular arrhythmia, severe aortic regurgitation, aortic annular rupture, and aortic dissection [5]. In general, hemodynamic instability is the most important situation for emergency ECMO implantation in the TAVI procedure. In their report, the need for ECMO for peri-procedural complication remained at 1%, regardless of the support of expertise. For our patient, the indication of ECMO was not because of complications from the TAVI procedure, but from the critical condition of AS. Her blood pressure fluctuated before the procedure of TAVI and dopamine was used to hold her vital signs.

The indication of prophylactic use of ECMO for AS patients with depressed left ventricular ejection fraction has also been studied. However, the outcome of patients appeared to not be improved [6]. Complications of ECMO may worsen the outcome. However, elective or prophylactic ECMO may be a feasible procedure to guarantee full hemodynamic capability during TAVI [7]. Our experience suggests that one indication of prophylactic use of ECMO is for the patients of an unstable hemodynamic condition. Further evidence may be needed to determine the necessity of using ECMO as a prophylactic or an emergent strategy [8].

## 4. Conclusions

The handling of patients with severe aortic stenosis and critical coronary artery disease is difficult. Especially in situations with unstable hemodynamics, a multidisciplinary team approach is crucial. The option of an emergency ECMO therapy has to be on site for coronary and structural interventions. To the best of our knowledge, we present the first case of a successful rescue-TAVI in Taiwan. This experience suggests prophylactic use of ECMO for patients with an unstable hemodynamic condition.

## Data Availability

The data used to support the findings of this study are available from the corresponding authors upon request.
